# 3-D Nucleus Architecture in Oat × Maize Addition Lines

**DOI:** 10.3390/ijms21124280

**Published:** 2020-06-16

**Authors:** Dominika Idziak-Helmcke, Tomasz Warzecha, Marta Sowa, Marzena Warchoł, Kinga Dziurka, Ilona Czyczyło-Mysza, Edyta Skrzypek

**Affiliations:** 1 Institute of Biology, Biotechnology, and Environmental Sciences, University of Silesia in Katowice, Jagiellońska 28, 40-032 Katowice, Poland; marta.sowa@us.edu.pl; 2 Department of Plant Breeding, Physiology, and Seed Science, University of Agriculture in Kraków, Podłużna 3, 30-239 Kraków, Poland; tomasz.warzecha@urk.edu.pl; 3 Department of Biotechnology, The Franciszek Górski Institute of Plant Physiology, Polish Academy of Sciences, Niezapominajek 21, 30-239 Kraków, Poland; m.warchol@ifr-pan.edu.pl (M.W.); k.dziurka@ifr-pan.edu.pl (K.D.); i.czyczylo@ifr-pan.edu.pl (I.C.-M.); e.skrzypek@ifr-pan.edu.pl (E.S.)

**Keywords:** addition line, *Avena sativa* L., chromosome territory, GISH, hybrid, nucleus, *Zea mays* L.

## Abstract

The nucleus architecture of hybrid crop plants is not a well-researched topic, yet it can have important implications for their genetic stability and usefulness in the successful expression of agronomically desired traits. In this work we studied the spatial distribution of introgressed maize chromatin in oat × maize addition lines with the number of added maize chromosomes varying from one to four. The number of chromosome additions was confirmed by genomic in situ hybridization (GISH). Maize chromosome-specific simple sequence repeat (SSR) markers were used to identify the added chromosomes. GISH on 3-D root and leaf nuclei was performed to assess the number, volume, and position of the maize-chromatin occupied regions. We revealed that the maize chromosome territory (CT) associations of varying degree prevailed in the double disomic lines, while CT separation was the most common distribution pattern in the double monosomic line. In all analyzed lines, the regions occupied by maize CTs were located preferentially at the nuclear periphery. A comparison between the tissues showed that the maize CTs in the leaf nuclei are positioned closer to the center of the nucleus than in the root nuclei. These findings shed more light on the processes that shape the nucleus architecture in hybrids.

## 1. Introduction

It is widely believed that the 3-D arrangement of interphase chromatin has a high impact on the functional response of the nucleus to the current needs of a cell. One of the basic features of nuclear architecture is its compartmentalization into distinct territories occupied by individual chromosomes [1,2]. The interior of the chromosome territories (CTs) is permeated by interconnecting channels that make it accessible for various regulatory factors, while intermingling at the borders of neighboring CTs allows for interchromosomal interactions [3,4]. The spatial positioning of chromosome territories greatly influences the key processes that take place in the nucleus, such as transcription, gene activity regulation, and DNA repair [5,6]. Still, despite the numerous studies, the mechanisms responsible for establishing the positions of the CTs in the nuclear space are not yet fully understood. Human and animal studies provided evidence that factors such as gene density [7], chromosome size [8], GC content [9], and the type of tissue [10,11] play a role in determining the position of the CTs relative to the nuclear center and to each other. Moreover, certain patterns of CTs distribution seem to be conserved among vertebrates [12,13]. In comparison to animals and human, the determinants of CTs arrangement in plant nucleus are far less known, although genome size [14], tissue specificity [15], endoreduplication [16], or geometrical constraints of the nucleus shape [17] were indicated to influence the nucleus architecture. Unfortunately, specific features of plants, such as the presence of the cell wall and various autofluorescence-generating cellular compounds, large genome sizes, and a high content of ubiquitous repetitive DNA sequences, pose significant challenges to investigating the plant nucleus in situ and in vivo [18]. Due to these obstacles, studies in plants are considerably scarce in comparison to the animal field. In particular, little is known about the arrangement of the parental genomes in allopolyploids and hybrids. Only a few reports indicate that they do not intermingle, but rather occupy distinct domains both during the interphase [19] and throughout the cell division [20,21]. A recent study on the spatial nucleus architecture in various wheat × rye and wheat × barley introgression lines demonstrated that the introgressed chromosome/chromosome arm homologs tend to occupy discrete, separate positions and rarely associate with each other in different somatic tissues and during different cell cycle stages [22]. The same study described the apparent link between the length of a chromosome and its spatial positioning, with shorter chromosomes or chromosome arms being preferentially located closer to the center of the nucleus and the longer chromosomes and chromosome arms occupying more peripheral areas of the nucleus.

In hybrids, the union of multiple genomes within one nucleus, concomitant with an increase in the DNA content, chromosome number, and ploidy level, requires establishing a new spatial arrangement of the former progenitor chromosomes. In some cases, the shock caused by this novel genomic constitution results in partial or complete uniparental chromosome loss when the chromosomes of one parent are preferentially eliminated during very early stages of embryo development [23,24,25,26,27]. 

Other processes that operate above the DNA level and lead to the stabilization of a newly formed hybrid nucleus include intergenomic chromosomal exchanges and homeolog losses that are random with respect to parental genome [28]. The intergenomic chromosome translocations occur frequently in hybrids or allopolyploids and affect, among other things, the meiotic behavior of the progenitor chromosomes and, consequently, fertility [29,30]. It has been demonstrated that non-random chromosome positioning and spatial proximity of CTs in the interphase nucleus influences the frequency of chromosome translocations [6,11]. Thus, the relative positions of the parental CTs might indirectly influence proper chromosome pairing during meiosis. Moreover, it has been demonstrated that the abnormal spatial positioning of certain chromosomal domains, such as telomeres, can reduce the ability of the alien chromosomes to pair properly in meiosis and lead to their gradual elimination over generations [31]. Taking these findings into account, the spatial arrangement of interphase chromatin in hybrid nuclei seems to have implications for the genetic behavior of the hybrid plants. Therefore, the basic research of nucleus 3-D architecture should be of great interest for plant breeders. 

Our project addresses this interesting topic by using a molecular cytogenetic approach to study the nucleus architecture in the oat × maize addition (OMA) lines with varying number of added chromosomes. Oat and maize are among the most distantly related plant species that could be sexually hybridized and produce fertile hybrids. In the wide crosses between oat and maize, uniparental elimination of maize chromosomes at the beginning of embryogenesis results in euhaploid oat plants or, occasionally, aneuhaploid plants with one or more maize chromosomes added to the oat genomic background [24,26]. In such plants, partial self-fertility results from meiotic restitution, which enhances the formation of unreduced gametes, and allows for the transmission of the added maize chromosomes [24,26,32]. Besides their potential utility in breeding programs, the OMA lines also provide valuable material for maize genomic studies. They were used for physical mapping of various genetic markers [33,34], analyses of gene expression [35,36,37], and studies on centromere structure and function [38,39] as well as meiotic chromosome behavior [40].

We have recently developed a series of novel, fertile OMA lines, with the added chromosome number varying from one to four. The OMA lines were produced from the sexual crosses between various oat genotypes (2 *n* = 42) and maize cv ‘Waza’ (2 *n* = 20) via embryo rescue. The chromosomes of the plantlets were doubled by colchicine treatment. Recovered OMA lines were characterized with regard to their phenotype, vigor, effectiveness in seed production, and the number of added chromosomes [41]. 

From these previously obtained lines, we selected several OMA plants with various numbers of added maize chromosomes in order to study the nucleus spatial architecture in partial hybrids. Using fluorescence in situ hybridization (FISH), confocal laser scanning microscopy, and state-of-the-art image analysis to perform nucleus 3-D reconstructions, we aimed to address the following questions: (1) is there a preferential positioning of the alien chromosomes in the nucleus of the recipient? (2) what are the factors that determine the position of the alien chromosomes? 

## 2. Results

### 2.1. Number and Identification of Maize Chromosome Additions

The plants used in the experiments belonged to the F2 generation of the OMA lines that had been obtained by wide crossing various oat genotypes with maize cv. ‘Waza’. For the purpose of this study, four OMA lines were selected: STH 6.8636, STH 5.8436 b, STH 6.8661, and STH 4.4690 p. All OMA lines possessed a complete set of oat chromosomes (2 *n* = 42). The presence of maize chromatin introgressions in those lines has been previously detected by PCR using primers that are specific for highly repetitive maize retrotransposon *Grande-1*, which is dispersed on all maize chromosomes [41]. In order to verify the number of maize chromosomes added to the oat genome in each of the lines, genomic in situ hybridization (GISH) has been performed on root-tip preparations using whole maize genomic DNA (gDNA) as a probe. A differentially labeled 25 S rDNA sequence was also used for GISH as an additional chromosome marker. The specific amplification of *Grande-1* indicates not only the presence of whole chromosome additions, but also small chromatin introgression that are undetectable by the GISH level of resolution. Thus, using 25 S rDNA as an additional probe allows for validating GISH quality in the case of the absence of maize gDNA signals.

**Figure 1:**
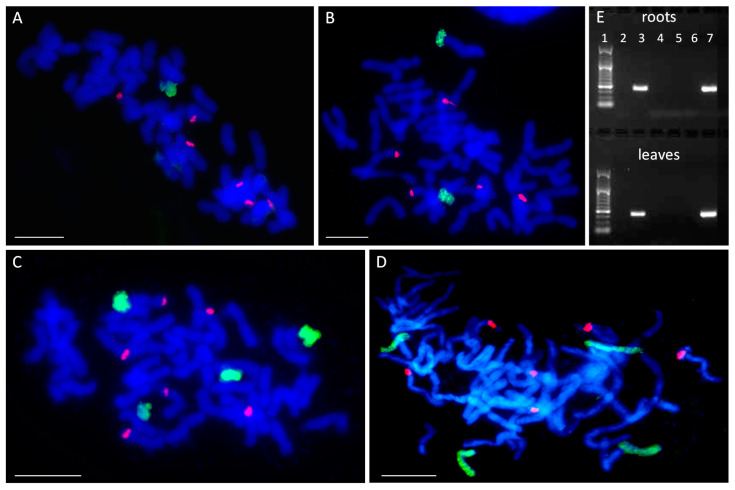
Number and identification of maize chromosome additions. (**A–D**) Visualization of added maize chromosomes in oat genome by genomic in situ hybridization (GISH). (**A**) STH 6.8636, 2 *n* = 43 (42 + Zm5′); (**B**) STH 5.8436 b, 2 *n* = 44 (42 + Zm3′ + Zm8′); (**C**) STH 6.8661, 2 *n* = 46 (42 + Zm3″ + Zm9″); (**D**) STH 4.4690 p, 2 *n* = 46 (42 + Zm1″ + Zm2″). Green fluorescence: maize genomic DNA (gDNA); red fluorescence: 25 S rDNA; blue fluorescence: DAPI. Scale bar: 10 µm. (**E**) Identification of added maize chromosomes by simple sequence repeat (SSR) markers. A representative agarose gel with bands representing DNA fragments that were amplified with SSR marker phi080 specific for maize chromosome 8. The marker leader is shown in lane 1. Maize specificity is shown by product presence in maize DNA (lane 3) and absence in oat DNA (lane 2). Chromosome 8 is absent from the lines STH 6.8636, STH 6.8661 and STH 4.4690 p (lanes 4, 5, and 6, respectively), and present in the line STH 5.8436 b (lane 7).

GISH detected the presence of one added maize chromosome in line STH 6.8636 (2 *n* = 43) (Figure 1A) and two maize chromosomes in STH 5.8436 b (2 *n* = 44) (Figure 1B). For future reference in this study, these lines were named I and II, respectively, with the Roman numerals reflecting the number of added chromosomes (see Table 1). The lines STH 6.8661 and STH 4.4690 p each had four maize chromosomes (2 *n* = 46) (Figure 1C,D) and have been renamed IVa and IVb, respectively (Table 1). All six 25 S rDNA loci were detected on oat chromosomes by the 25 S rDNA probe. However, none of the added maize chromosomes in any of the lines carried a 25 S rDNA sequence (Figure 1).

Next, the added maize chromosomes were identified in the analyzed OMA lines by PCR with the simple sequence repeat (SSR) markers specific for particular maize chromosomes [24,32]. An SSR marker was considered suitable to use in chromosome identification if a single PCR product was specifically amplified from maize cv. ‘Waza’ gDNA and no product was detected from the gDNA of oat cv. ‘Stoper’ (Figure 1E). The final list of SSR markers used for chromosome identification is presented in Table 2.

The results of the PCR analyses indicated that OMA line I comprises chromosome 5 of the maize genome (2 *n* = 42 + Zm5′), while line II is a double monosomic characterized by the presence of maize chromosomes 3 and 8 (2 *n* = 42 + Zm3′ + Zm8′). Figure 1E shows a representative agarose gel with PCR product corresponding to maize chromosome 8 present in OMA line II. Both lines with four chromosome additions were found to be double disomics. Line IVa has a pair of chromosomes 3 and a pair of chromosomes 9 (2 *n* = 42 + Zm3″ + Zm9″), whereas line IVb contains a pair of chromosomes 1 and a pair of chromosomes 2 (2 *n* = 42 + Zm1″ + Zm2″) (Table 1). The identification of added maize chromosomes was performed on gDNA isolated from root and leaf tissue. However, no differences between the tissues were discovered.

### 2.2. Size and Associations of Maize Chromosome Territories

**Figure 2:**
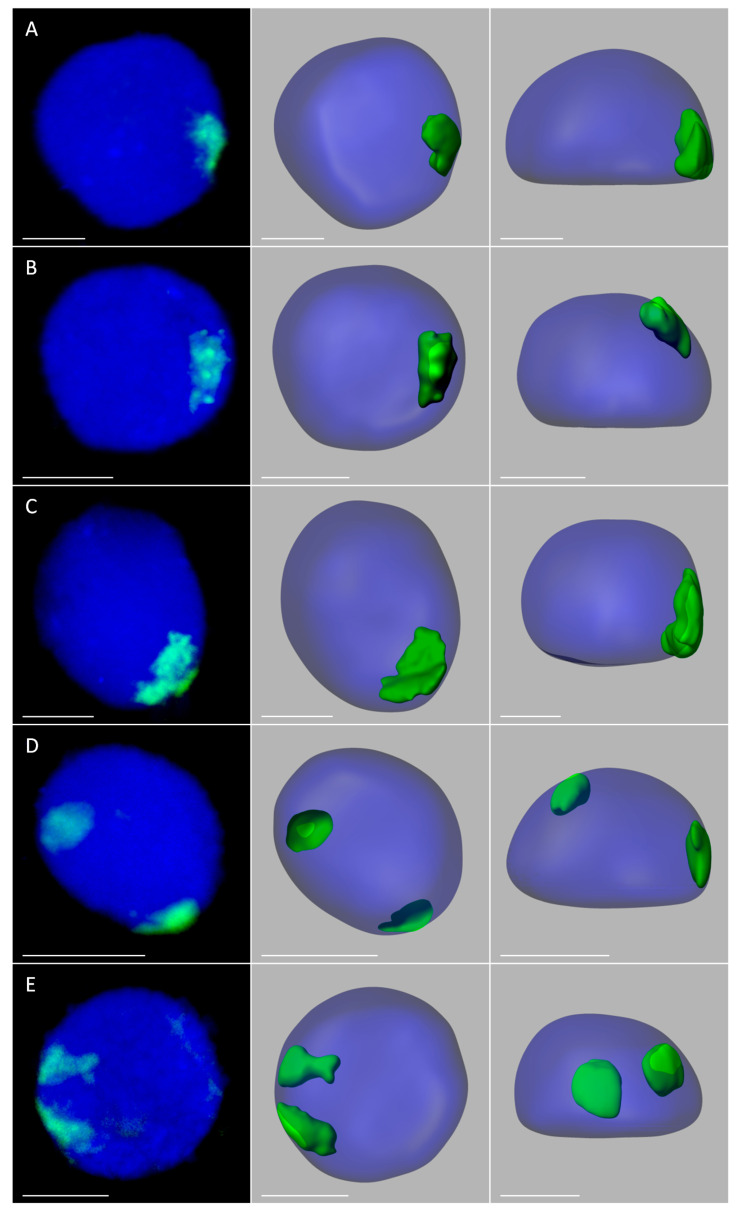
Maize chromosome territories (CTs) in the oat background; OMA lines with one (**A**,**B**) and two (**C**–**E**) maize chromosome additions. The left column shows GISH results with maize gDNA as a probe (green fluorescence). Chromatin is stained with DAPI (blue fluorescence). The middle and right columns present the models of nuclei and maize CTs observed in two different planes. (**A**) line I, root; (**B**) line I, leaf; (**C**) line II, root, association of maize CTs, (**D**) line II, root, separation of maize CTs; (**E**) line II, leaf, separation of maize CTs. Scale bar: 2 µm.

**Figure 3:**
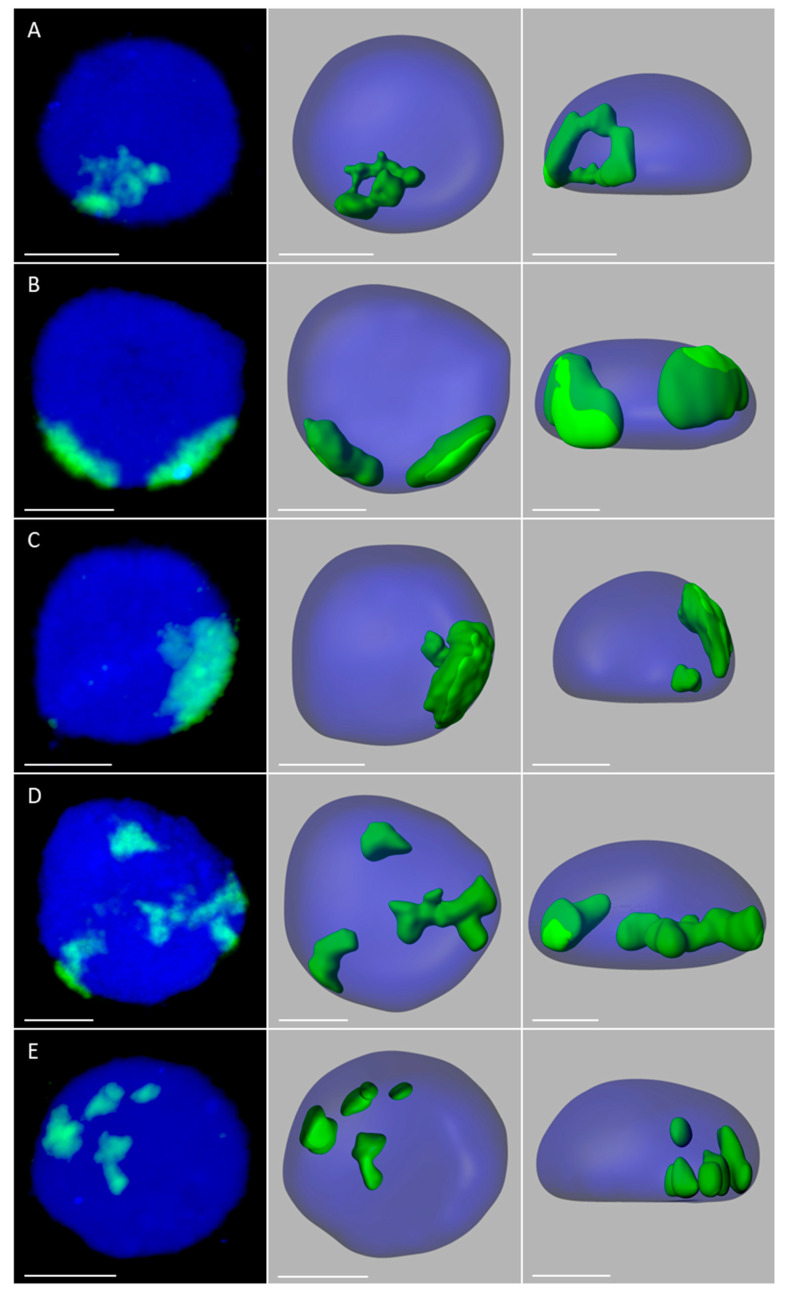
Maize chromosome territories (CTs) in the oat background; OMA lines with four maize chromosome additions. The left column shows GISH results with maize gDNA as a probe (green fluorescence). Chromatin is stained with DAPI (blue fluorescence). The middle and right columns present the models of nuclei and maize CTs observed in two different planes. (**A**) line IVa, root, full association of maize CTs; (**B**) line IVb, root, two maize chromatin areas of similar size; (**C**) line IVb, root, two maize chromatin areas of different size (**D**) line IVb, leaf, three maize chromatin areas; (**E**) line IVa, root, total separation of maize CTs. Scale bar: 2 µm.

**Figure 4:**
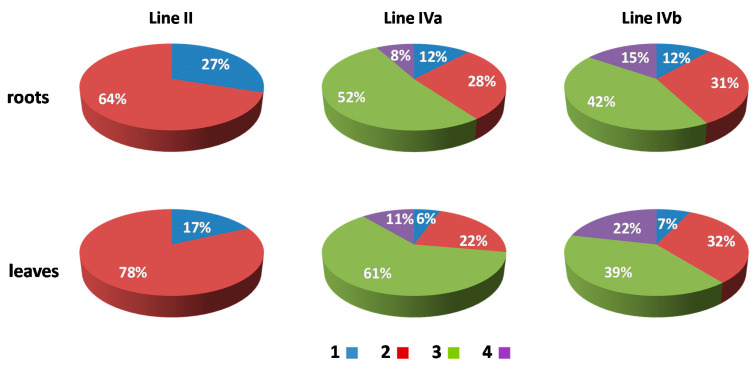
The association frequencies of added maize chromosomes in the root and leaf nuclei of OMA lines with two (line II) and four (lines IVa and IVb) chromosome additions. Blue (1): one area containing maize chromatin (total association of maize CTs); red (2): two areas containing maize chromatin; green (3): three areas; purple (4): four areas (total separation of maize CTs).

In order to analyze the size, position, and possible associations of territories formed by maize chromosomes added to the oat genome, GISH with maize gDNA as a probe was performed on isolated nuclei from leaf and root tissues of the four OMA lines. Three morphological types of the nuclei were observed in the preparations: spherical, elongated, and rod-like. However, for the purpose of this study only the spherical and slightly elongated nuclei were analyzed. The maize chromosomes formed distinct, cloud-like territories, frequently located at the nuclear periphery (Figure 2 and Figure 3, Supplementary Videos S1–S10). The peripheral positioning of a maize CT was predominant both in the leaf and root nuclei of line I, with maize chromatin often protruding out from the nucleus chromatin bulk (Figure 2A,B, Supplementary Videos S1 and S2). In line II, the two added maize chromosomes usually formed two separate territories, often located on the opposite sides of the nucleus (Figure 2D,E, Supplementary Videos S4 and S5). The association of those two CTs was also observed (Figure 2C, Supplementary Video S3), both in root and leaf nuclei, with the association frequencies of 27% and 17%, respectively (Figure 4). A similar situation occurred in another OMA line STH 4.4576 (2 *n* = 44) (not included in the present paper due to the difficulties associated with reliable chromosome identification) with two maize chromosome additions. In that line the maize CTs were also mostly separated and the association frequencies equaled 17% in the roots and 11% in the leaf nuclei.

The number of areas occupied by maize chromatin in the nuclei of OMA lines IVa and IVb ranged from one to four (Figure 3, Supplementary Videos S6–S10). The total separation of the CTs occurred in 8% of the root nuclei in line IVa and 15% of the root nuclei in line IVb (Figure 4), indicating that the CTs association is the preferred maize chromatin distribution pattern. In the leaf tissue, the separation of CTs was more frequent with 11% in line IVa and 20% in line IVb. Still, chromosome associations of varying degree prevailed in the leaf nuclei. The four CTs typically differed in size (Figure 3E), with one territory being usually much larger than the other ones. This observation suggests that the condensation level might differ significantly between the maize homologs. In both lines, independent of tissue type, the most commonly found association pattern was characterized by the presence of three areas containing maize chromatin (Figure 4). The question, whether the area formed by two associated CTs comprises homologous or heterologous chromosomes, however, cannot be answered through the present GISH experiments and should be the subject of further analyses. When the added maize chromosomes formed two regions in the nucleus, two situations were discerned. In the first one, both areas were similar in size (Figure 3B), indicating that each one comprised two chromosomes. In the second case, the two areas clearly differed in size (Figure 3C), which allows for the assumption that the larger area contained three chromosomes, and the fourth chromosome formed a separate CT. The full association of maize CTs, indicated by the presence of only one area containing maize chromatin, was found in only 12% of IVa and IVb root nuclei and 6% and 7% of IVa and IVb leaf nuclei, respectively.

The size and association frequency of chromosome territories formed by the added maize chromosomes were analyzed in each OMA line using a sample of ca. 20–30 nuclei that were randomly selected from root and leaf tissue preparations. The 3-D models of the nuclei and maize CTs were utilized to determine the volume of the nucleus (V_nuc_), the volume of individual maize CTs or individual regions occupied by associated maize CTs (V_Zm_), as well as the sum of the volumes of all maize CTs in a given nucleus (V_tot_). In order to allow for the comparison between the lines without the bias caused by the differences in nuclei sizes, the V_Zm_ and V_tot_ values were calculated also as a percentage of the nuclear volume. The maximum, minimum, average, median, and standard deviation values of V_nuc_, V_Zm_, and V_tot_ for both types of tissue in the four OMA lines are presented in Table 3. 

**Figure 5:**
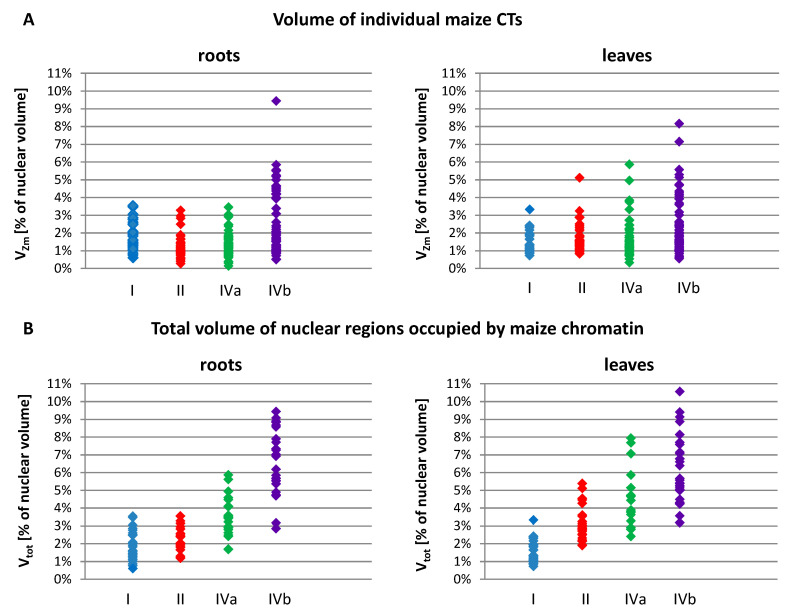
The size of the regions occupied by maize chromosome additions in the root and leaf nuclei of OMA lines with one (line I), two (line II), and four (lines IVa and IVb) added chromosomes. (**A**) The volumes of individual maize CTs or regions occupied by associated maize CTs (V_Zm_). (**B**) The total volumes of all maize CTs present in a given nucleus (V_tot_). The volume values are calculated as a percentage of the nuclear volume.

The nuclear volume ranged from 238 µm^3^ in the leaves of OMA line IVa to 1849 µm^3^ in the leaves of line I. Among all OMA lines, the average size of leaf nuclei was consistently smaller than the average size of root nuclei. We noticed that in the lines with four maize chromosome additions, particularly line IVa, the average and median nuclear sizes were smaller than in the lines with one or two added maize chromosomes. In terms of the size of individual maize CTs, as well as the sum of their volumes in a nucleus, the OMA lines with four chromosome additions differed considerably from each other (Table 3, Figure 5). Line IVa was characterized by the lowest average volume of individual maize chromosome territory (V_Zm_av_), both in roots and leaves. However, when V_Zm_av_ values were calculated as a percentage of nuclear volumes, the results obtained for line IVa were similar to those obtained for lines I and II. In contrast, these values for line IVb were the highest among the analyzed OMA lines. Figure 5A presents the range of V_Zm_ values (as a percentage of the nuclear volume) in all lines. The volumes of individual maize CTs were similar in lines I, II, and IVa, and varied from 0.14% to 3.53% in root nuclei and from 0.33% to 5.86% in leaf nuclei. In line IVb these ranges were wider, reaching 9.44% and 8.16% for root and leaf nuclei, respectively. These numbers indicate that an individual maize CT can occupy a larger portion of the nuclear space in line IVb than in the other lines.

The total sum of the volumes of all maize CTs present in the nucleus (V_tot_) increased, as could be expected, with the increasing number of maize chromosomes added to the oat genome (Figure 5B). Nevertheless, there were striking differences between the two lines with four maize chromosomes. In line IVa, the average values (V_tot_av_) equaled 3.47% in root nuclei and 4.56% in leaf nuclei, whereas in line IVb the maize chromatin occupied on average 6.53% and 6.15% of the nuclear volume in roots and leaves, respectively. The minimal and maximal values of V_tot_ were also higher for line IVb in both types of tissue. This observation indicates that, beside the number of added chromosomes, the type of chromosome also plays a role in determining the amount of space that is taken up by the introgressed chromatin in the host nucleus.

**Figure 6:**
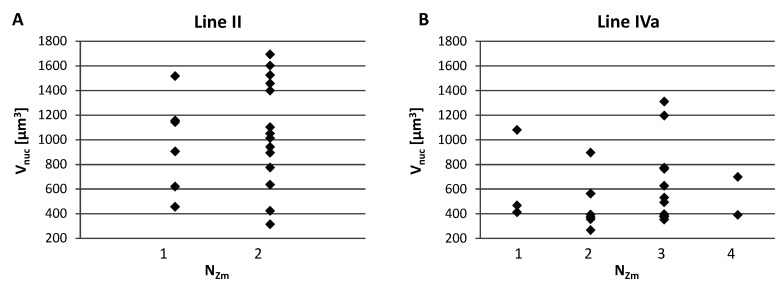
Representative charts demonstrating the number of regions formed by single or associated maize CTs (N_zm_) in the root nuclei of varying size. (**A**) Line II with two maize chromosome additions. (**B**) Line IVa with four maize chromosome additions. The maize chromosomes may form various associations regardless of the nucleus size.

**Figure A1:**
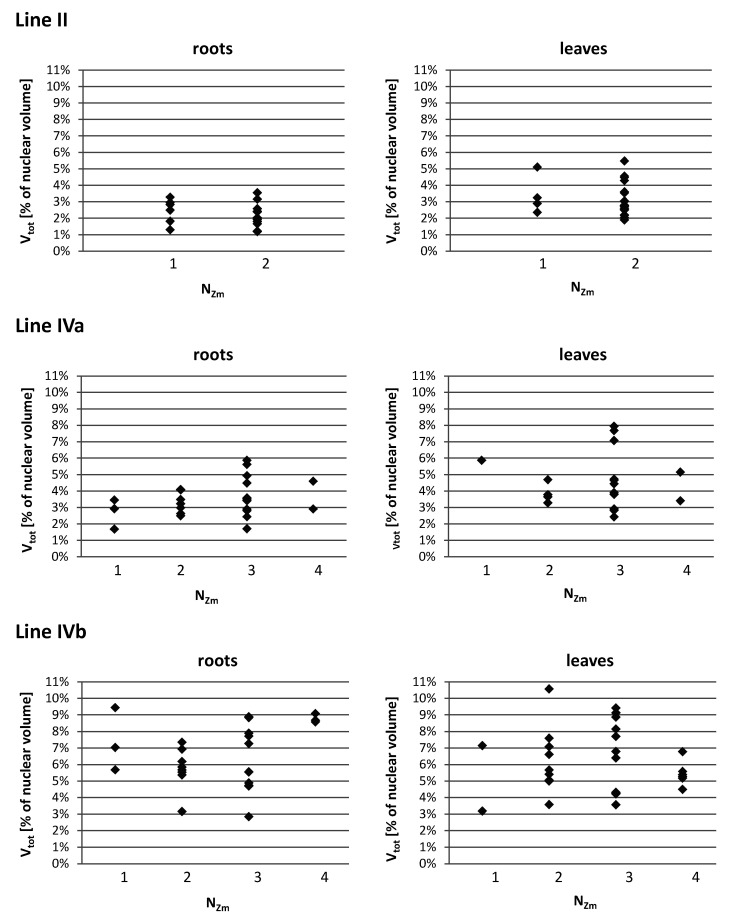
The ranges of the total volume of maize chromatin V_tot_ (expressed as the percentage of nuclear volume) in the nuclei with varying number of regions formed by single or associated maize CTs (N_zm_). The V_tot_ varies within the same range regardless of the CTs association pattern.

We analyzed possible correlations between the number of areas formed by the maize CTs inside the nucleus (N_Zm_), the nucleus size (V_nuc_), and the total volume of maize chromatin (V_tot_). The assessment of the relationship between the V_nuc_ and N_Zm_ indicated that the maize chromosomes may form various associations regardless of the nucleus size, as shown by representative charts presented in Figure 6. The low values of the Spearman’s rank correlation coefficients calculated for V_nuc_ and N_Zm_ confirmed the hypothesis that the number of regions occupied by maize chromatin does not depend on the nuclear volume. Analogous analyses showed that there is no correlation between N_Zm_ and V_tot_. This means that the total volume that the maize CTs occupy in the hybrid nucleus varies within the same range, regardless of their association pattern (Figure A1).

Finally, the relationship between V_nuc_ and V_tot_ was examined. A comparison between the average and median values of V_tot_ showed that the median (V_tot_med_) was lower than the average (V_tot_av_) in all studied OMA lines in both tissues (Table 3), which indicated a positively skewed distribution of V_tot_. Due to the asymmetrical distribution, the commonly used Pearson linear correlation could not be applied, so, Pearson’s χ^2^ test was used instead to verify the hypothesis of the independence of the variables. It demonstrated that both variables are independent at the significance level α = 0.05.

### 2.3. Positioning of Maize Chromatin-Occupied Regions

**Figure 7:**
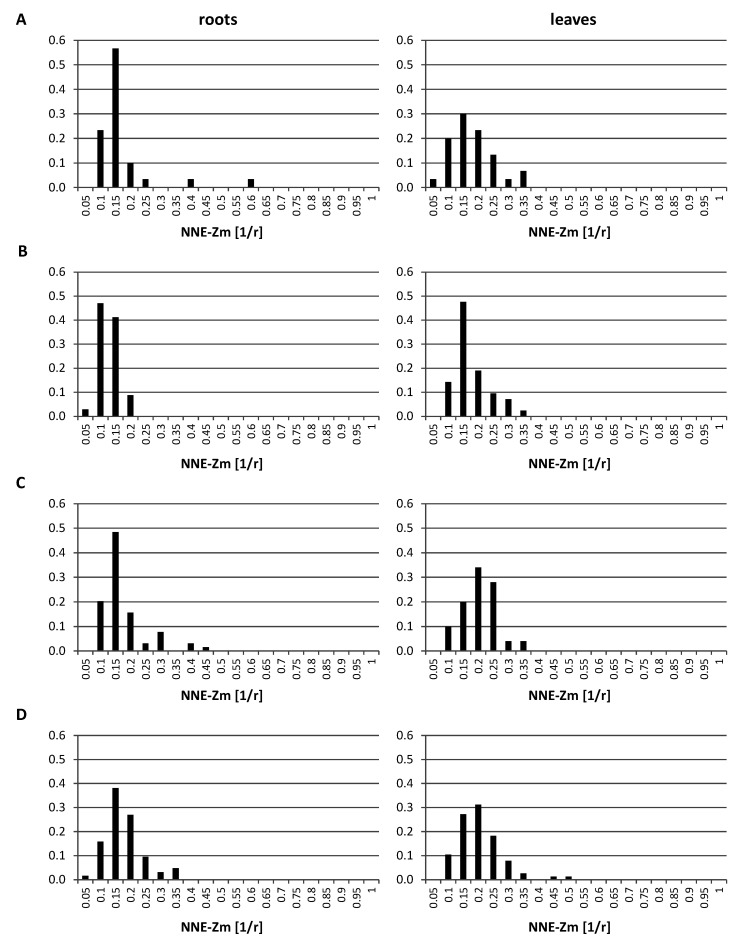
The probability distribution of nearest nuclear edge (NNE-Zm) distances in the root and leaf nuclei. (**A**) line I; (**B**) line II; (**C**) line IVa; (**D**) line IVb. The NNE-Zm distances were normalized by the cube root of nuclear volume, which allowed for analyzing them as fractions of the nuclear radius *r*.

The analysis of the GISH data indicated that the regions occupied by single or associated maize CTs locate preferentially at the nuclear periphery (Figure 2 and Figure 3). In order to examine the positioning of maize chromosomes in a quantitative way, the distances between the centers of the regions formed by maize chromatin and the nearest nuclear edge (NNE-Zm) were measured using the “Measurement Points” function of Imaris. The distances were normalized by the cube root of the nuclear volume, which allowed us to treat them as fractions of the nuclear radius *r*. The probability distribution of those distances was estimated for all maize chromatin regions in each OMA line in both tissues. The probability distributions presented in Figure 7 confirm the preferential positioning of maize chromosomes close to the edge of the nucleus. The results for all the lines were similar, although the modes of the distribution for the lines with four added chromosomes suggested that their maize CTs tend to locate slightly farther away from the nuclear envelope than in the other OMA lines. The comparison between the tissues showed that in lines II, IVa, and IVb, the maize CTs in the leaf nuclei are positioned closer to the center of the nucleus than in the root nuclei. The modes of the probability for the leaf tissue in those lines were shifted toward the nucleus center by 0.05 *r*. Moreover, the probability distributions in the leaf nuclei were wider than in the root nuclei, indicating more variation of the NNE-Zm distance in the leaves.

**Figure 8:**
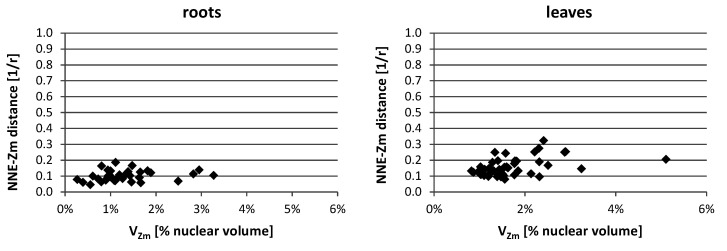
Representative charts demonstrating the NNE-Zm distances of the maize chromatin-occupied regions with varying size in the nuclei of line II. No correlation could be observed between the size of individual or associated maize CTs (V_Zm_) and their distance from the nucleus edge.

In the lines with more than one added maize chromosome, the influence of the maize chromosome associations on the NNE-Zm values was also evaluated. The analysis of probability distributions, when they were calculated separately for nuclei with differing N_Zm_ (data not shown), indicated that the number of maize chromatin-occupied regions in a nucleus did not play a role in determining their position. Additionally, the average NNE-Zm distances calculated for nuclei with varying number of maize chromatin regions did not correlate with that number. Likewise, no correlation was found between the size of a region occupied by maize chromatin (V_Zm_) and its distance from the nuclear edge (Figure 8).

## 3. Discussion

Interspecific and intergeneric crossing between cultivated plants or cultivated plants and their wild relatives represents the most basic yet the most powerful tool with which plant breeders may obtain genetically improved crops. Broadening the gene pool through the introgression of alien chromatin to the crop cultivars can enhance desired agronomic traits and improve tolerance to biotic and abiotic stress [42]. In wide crosses, a frequent phenomenon is either complete or partial uniparental chromosome elimination, which results in the production of haploid plants or chromosome addition lines, respectively, both outcomes being valuable for breeding purposes as well as basic research. Wide crosses between Panicoideae and Pooideae have long attracted researchers’ and plant breeders’ interest as a source of genetic stocks for both genomic studies and crop improvement, with particular focus on oat and maize hybridization, since their hybrid embryos are able to grow into partially fertile plants [43]. In various oat × maize addition lines, the expression of maize genes was shown to introduce some C4 photosynthesis characteristics into oat and to impact oat morphological features [36,37,44], indicating the potential for transferring beneficial maize traits to oat. OMA lines significantly contributed to advances in the physical mapping of the maize genome [34,45] and studies on the transcriptional and epigenetic adaptation of maize genes in new genomic environments [35]. 

The utility of addition lines is entirely based on the stable maintenance of the introgressed chromatin in the successive generations. It has been suggested that the retention of the added chromosomes might be dependent on their position in the hybrid nucleus. A recent study indicated that improper positioning of the introgressed alien chromosomes in nuclei may negatively affect their ability to migrate into the leptotene bouquet at the onset of meiosis, resulting in pairing failure and, consequently, in the loss of such chromosomes [31]. In wheat × rye introgression lines, the frequency of rye telomere misplacement in somatic nuclei was almost identical to the frequency of out-of bouquet rye telomere position in leptotene, indicating that there may be a systemic failure of some rye telomeres to assume proper positions in the wheat × rye nucleus [46]. Analysis of seven various wheat × rye introgression lines revealed that chromosome conformation is one of the factors that influenced the frequency of defective migration of rye telomeres into the bouquet [47,48]. Another proposed consequence of the out-of-position placement of the rye telomeres is reduced rye chromosome pairing in meiotic metaphase I [46], which is thought to be responsible for aneuploidy observed in triticale hybrids [49]. Moreover, it is known now that direct physical interchromosomal interactions are involved in transcriptional regulation, suggesting a strong link between the gene expression and the position of a CT relative to other CTs within the nuclear space [3,5,50]. Based on these discoveries, one can presume that the spatial position of introgressed chromatin in the nucleus of a recipient species will greatly influence both the genetic stability of an addition line and its usefulness in successful transfer of agronomically desired traits to cultivated crops. 

This assumption prompted us to investigate the 3-D spatial arrangement of maize added chromosomes in four OMA lines with various numbers of chromosome additions. In this study, we focused on the F2 generation of OMA plants. This posed a methodological challenge due to the extremely limited amount of plant material to study, which was caused by a very low number of seeds produced by the F1 plants. Because of these limitations, we could not apply the methods that would require plenty of tissue to ensure proper nuclei density in preparation, such as e.g., embedding the nuclei in polyacrylamide gel. Our future research will comprise next generations of OMA lines, which should allow us to overcome the shortcomings resulting from the limited availability of plant material and should also provide information about the transmission rate of added chromosomes in relation to their spatial arrangement. 

We found out that the association of added maize chromosomes is a preferred arrangement pattern in the double disomic lines IVa and IVb. Whether the associated chromosomes are homo- or heterologs remains to be determined and will be an important part of our future research. Although it would seem that homolog association is favorable for the cell, since it could facilitate DNA repair processes via homologous recombination [6], many studies point out that this is not the case. Analysis of mammalian cells showed that distances between the homologs in the nucleus are generally larger that the distances between heterologs [51]. In somatic nuclei of *Arabidopsis thaliana* and *A. lyrata*, the spatial distribution of the chromosomes is predominantly random, and the only associations that are more frequent than those predicted by the random spatial distribution simulations are those involving nucleolar organizing region (NOR)-bearing chromosomes, which in most nuclei are attached to a single nucleolus [52,53]. In various wheat × rye and wheat × barley introgression lines, the complete separation of introgressed homologs in somatic nuclei was the most frequently observed arrangement, independent of the cell cycle stage or tissue type [22]. In contrast, in wheat × barley substitution lines, the association of barley homologs was found in the pre-meiotic cells, as well as in all the surrounding somatic tapetum cells [54]. The preferential pairwise association of one maternal and one paternal homolog was described in the triploid endosperm nuclei of *A. thaliana* [15]. Moreover, in the grass *Brachypodium distachyon*, homologous chromosome arm CTs in root nuclei were shown to associate more frequently than expected at random [55]. Such variation in the data obtained from various species does not allow us at present to make any assumptions about the rate of homo- or heterologous associations in the double disomic OMA lines. Addressing this question would require the identification of added maize chromosomes in the 3-D preserved nuclei by fluorescence in situ hybridization (FISH) with specific chromosomal markers. The discrimination of maize chromosomes was accomplished previously using homo- or heterologous mapping of bacterial artificial chromosome (BAC) clones [56] or repetitive DNA sequences [57]. Recently, whole-chromosome oligo-FISH paints using synthetic oligonucleotide libraries have been developed for all 10 chromosomes of maize, providing a powerful tool kit for cytomolecular studies [58].

In oat interphase nuclei, the chromosomes assume a polarized orientation with centromeres grouped on one side of the nucleus and telomeres occupying the opposite nuclear pole, known as the Rabl orientation [59,60]. In plants, such organization of interphase chromatin has been usually attributed to species with large genomes of more than 4800 Mb, such as oat, wheat, barley, and rye. Small genome plants, like rice, sorghum, Arabidopsis, and Brassica do not display the Rabl-like arrangement [14,61]. However, there are deviations from this pattern, as the presence of the Rabl configuration was described in the root nuclei of *B. distachyon* with the genome size of 300 Mb [17]. Maize, with intermediate genome size (~3000 Mb), does not assume the Rabl configuration, although it was shown that the maize centromeres do not disperse uniformly in the entire volume of the nucleus, which is the case with other non-Rabl displaying plants [14]. 

In wheat × rye and wheat × barley disomic substitution and addition lines, the introgressed chromosomes assume the Rabl orientation alongside the chromosomes of a host species. They adopt a string-like configuration with chromosomal arms extending from the centromere pole to the telomere pole [22]. Such distribution could be expected since both the donor and the recipient species show the Rabl pattern in their native form. In contrast, the shapes of the maize CTs in oat × maize addition lines are strikingly different and resemble clouds rather than strings, indicating that they do not follow the Rabl pattern typical for oat. This might be due to the much smaller size of maize chromosomes compared to oat, which might hamper stretching them between the centromere and telomere poles. It is possible, too, that the mechanisms responsible for establishing and maintaining the Rabl configuration in oat fail to order maize chromosomes in a similar manner. Such mechanisms are largely unknown, although in fission yeast a role for a Csi1 protein and SUN domain protein Sad1 in centromere clustering has been proposed [62]. Similar to OMA lines, in the hybrid nuclei of wheat and pearl millet, which is also a non-Rabl plant, the pearl millet chromatin was found to occupy spherical or spindle shape territories, suggesting that in this case the Rabl orientation was also not imposed by the wheat host [23]. Whether the presence of added maize chromosomes affects the Rabl configuration of oat remains an open question. In our preliminary experiments of mapping the telomeres and centromeres to the nuclei of OMA lines, the polarized oat chromatin arrangement does not seem to be much disrupted, but more detailed studies addressing the influence of the number of chromosome additions on the oat nuclear architecture have to be conducted. Another interesting issue that needs examination is the positioning of maize centromere(s) and telomeres in the somatic and meiotic nuclei of OMA plants. Inferred from the findings of Pernickova et al. [31], telomere and centromere positioning can have implications for the ability of maize chromosomes to participate in the formation of the leptotene bouquet and consequently, the transmission rate of chromosome additions to the next generations of OMA plants.

Another similarity between oat × maize and wheat × pearl millet hybrid nuclei is the predominant localization of introgressed chromatin at the nuclear periphery. The peripheral positioning of pearl millet chromatin was shown to be part of a chromosome elimination pathway that involved the formation of nuclear extrusions in the interphase [23]. Additionally, in *Hordeum vulgare* and *H. bulbosum* hybrids, the *H. bulbosum* chromatin destined for elimination was deposited at the nuclear edge [63]. In our study, the maize CTs were often found protruding out from the nucleus. Although it seems that these protrusions do not lead to chromosome elimination in F2 OMA plants, whether it would affect the transmission rate of added chromosomes to subsequent generations of OMA lines is to be determined. It is tempting to speculate that confining maize or pearl millet chromosomes to the nuclear periphery is a part of preserving the Rabl orientation of oat and wheat chromosomes, respectively. This speculation is corroborated by the fact that no clear spatial separation of genomes was found in triticale, where both parental genomes display Rabl arrangement [22].

In our study we did not find any apparent correlations between the size of the maize chromatin-occupied areas, their number, and the size of the nucleus. Neither was the size of maize separated or associated CTs influencing their position within the nucleus. Obviously, the total volume of maize chromatin depended on the number of added chromosomes, but the chromosome content also seemed to play a significant role. The added chromosomes 1 and 2 in line IVb occupied much more nuclear space than chromosomes 3 and 9 in the other double disomic line IVa. It pertains to the fact that chromosomes number 1 and 2 are the largest in the whole chromosome complement of maize [64]. Based on the DNA content measurements in individual maize chromosomes (according to [64]), chromosomes 1 and 2 contain together 1.23 times more DNA than chromosomes 3 and 9. The ratio between the average total maize chromatin volume (V_tot_av_ [%]) values for lines IVb and IVa is 1.88 in the case of root nuclei and 1.34 for the leaf nuclei. This comparison shows that the increased volume of maize chromatin in line IVb cannot be explained by the differences in DNA content between the chromosome additions alone, and that other factors must play a role.

One of the possible reasons why we could not find correlations between the measured parameters is the fact that our research was conducted on the heterogeneous populations of nuclei. It cannot be excluded that the analyses performed on the nuclei segregated according to the cell cycle stage, DNA content, or localization in the plant organ, would yield different results. 

In our future work, which will comprise next generations of OMA lines, flow-sorting or digital image cytometry of the nuclei will be employed in order to analyze the nuclear architecture in oat × maize hybrids in a cell-cycle dependent manner. As already mentioned above, other topics to be investigated are the frequency of homo- versus heterologous associations of the added chromosomes as well as 3-D telomere and centromere distribution in the hybrids. The present study on F2 OMA plants thus serves as a basis from which various directions of prospective research can stem out.

## 4. Materials and Methods

### 4.1. Plant Material

The OMA lines used in this study have been selected from the F2 generation of OMA plants. The F1 OMA plants were obtained by wide crossing of various oat (*Avena sativa* L., 2 *n* = 42) genotypes with maize (*Zea mays* L. var. *saccharata*, 2 *n* = 20) cv. ‘Waza’ and subjected to colchicine treatment. The presence of maize chromatin introgressions in those lines was confirmed beforehand by PCR with primers that specifically amplify a maize retrotransposon *Grande-1* [41]. The following OMA lines were chosen for the present study: STH 6.8636, STH 5.8436 b, STH 6.8661, STH 4.4690 p. Oat cv. ‘Stoper’ and maize cv. ‘Waza’ were used as control. The seeds were germinated on moist filter paper in Petri dishes in the dark. After 3–4 days the seedlings were transferred to the perlite and soil mix. Plants were grown in the greenhouse at 21 °C with a 16 h photoperiod and light intensity of 100 µmol m^−2^ s^−1^.

### 4.2. Chromosome Preparations

Mitotic chromosome preparations of the root meristems were made according to a previously described procedure [65] with minor modifications. The seeds of oat-maize addition lines, oat cv. ‘Stoper’, and maize cv. “Waza” were germinated for 3–4 days in the dark in Petri dishes moistened with distilled water. Roots of 1.5–2 cm length were excised from the seedlings and incubated in ice-cold water for 24 h, then fixed in a mixture of methanol/glacial acetic acid in a 3:1 (*v*/*v*) proportion, and stored at −20 °C until use. Excised root tips were washed in 0.01 M citric acid/sodium citrate buffer (pH 4.8) for 15–20 min and digested in an enzyme mixture comprising 4% (*v*/*v*) pectinase (Sigma-Aldrich, St Louis, MO, USA), 1% (*w*/*v*) cellulase (Calbiochem, San Diego, CA, USA), and 1% cellulose ‘Onozuka R-10′ (Serva, Heidelberg, Germany) for 1.5 h at 37 °C. After digestion, the meristems were dissected from the root tips in a drop of 45% acetic acid, transferred onto the slide, covered with coverslips, and gently squashed. The slides were frozen on dry ice for 20–25 min. After freezing and removing the coverslips, the slides were air-dried and stored at 4 °C until used.

### 4.3. Isolated Nuclei Preparations

Root and leaf tissue for the isolated nuclei preparations were taken from 8–10 week-old plants. The nuclei isolation was done according to [66] with modifications. Each step of the following procedure was performed on ice. The collected tissue was fixed in 4% formaldehyde in PBS buffer (pH 7.3) for 30 min on ice, washed three times in PBS (10 min each time), and then washed in TRIS buffer (10 mM TRIS-HCl, pH 7.5, 10 mM Na_2_-EDTA, 100 mM NaCl) for 20 min. Next, the tissue was chopped with a razor blade in LB01 buffer (15 mM TRIS-HCl pH 7.5, 2 mM Na_2_-EDTA, 0.5 mM spermine 4 HCl, 80 mM KCl, 20 mM NaCl, 0.1% Triton X-100, 15 mM β-mercaptoethanol) in a Petri dish. The nuclei suspension was filtered through a mesh filter with a pore size of 30 µm and dropped onto poly-L-lysine covered microscopic slides that had been cooled down to 0 °C. In the case of leaf tissue, the suspension of nuclei was additionally centrifuged (700 *g*, 4 °C, 3 min) in 0.1% Triton X-100 in PBS to remove the chloroplasts and then the nuclei pellet was suspended in PBS. The slides were air-dried and stored at −20 °C until use.

### 4.4. DNA Extraction and PCR Analysis for Chromosome Identification

The total genomic DNA of oat-maize addition lines, oat cv. ‘Stoper’, and maize cv. “Waza” was extracted from roots and leaves of plants grown in a greenhouse (21 °C, 16 h photoperiod, light intensity of 100 µmol m^−2^ s^−1^). About 0.8 g of fresh tissue was frozen with liquid nitrogen and homogenized. DNA isolation was performed using DNeasy Plant Mini Kit (Qiagen, Hilden, Germany) according to maufacturer’s recommendations. The concentration of the isolated DNA was measured at 260 nm by NanoDrop 2000 c (Thermo Fisher Scientific, Waltham, MA, USA) and its quality was checked on agarose gel.

The added chromosomes in the studied OMA lines were identified using maize chromosome-specific SSR markers selected from the Maize Genome Database (https://www.maizegdb.org) and described before by [24] and [32]. The PCR with the primer sets for each SSR marker was performed on the leaf and root tissue OMA DNA and the DNA of oat cv. ‘Stoper’ and maize cv. ‘Waza’ was used as a negative and positive control, respectively. The PCR products were amplified with the following program: 94 °C/5 min → (94 °C/40 s → 63 °C/40 s → 72 °C/45 s) × 35 → 72 °C/10 min → 4 °C/∞. The obtained products were mixed with 6× *TriTrack DNA Loading Dye* (Thermo Fisher Scientific, Waltham, MA, USA) and separated in 1.5% agarose gel under 80 V for 60 min. The O’RangeRuler 50 bp DNA Ladder (Thermo Fisher Scientific, Waltham, MA, USA) was used to estimate the length of PCR products.

### 4.5. DNA Labeling and In Situ Hybridization

The maize cv. ‘Waza’ total genomic DNA was used in order to discriminate maize chromosomes in the oat background. Maize genomic DNA was isolated using DNeasy Plant Mini Kit (Qiagen, Hilden, Germany) according to manufacturer’s recommendations and labeled by nick-translation with digoxigenin-11-dUTP (Roche, Basel, Switzerland) as described by [67].

The in situ hybridization procedure was adopted from [65] with minor modifications. In brief, the slides were pre-treated with RNase, washed several times in a 2× saline sodium citrate (SSC) buffer, dehydrated in an ethanol series (70%, 90%, 100%), and air-dried. The DNA probes used in experiments (~500 ng/slide) were mixed together, precipitated, and dissolved in a hybridization mixture containing 50% deionized formamide, 10% dextran sulfate, 2× SSC, 0.5% SDS, and water. In the genomic in situ hybridization (GISH) experiments that involved maize total genomic DNA, the use of genomic oat DNA to block non-specific hybridization was not necessary due to the high phylogenetic distance between oat and maize. The hybridization mixture was predenatured (75 °C, 10 min), applied to slides, and denatured again at 75 °C for 4.5 min. Hybridization was performed in a humid chamber at 37 °C for about 40 h. After hybridization, the slides were washed in two changes of 10% formamide in 0.1× SSC (5 min each) at 42 °C, which is equivalent to a 79% stringency. The immunodetection of digoxigenin- and biotin-labeled probes was performed according to standard protocols using FITC-conjugated anti-digoxigenin antibodies (Roche, Basel, Switzerland). The preparations were mounted and counterstained in VectaShield antifade (Vector Laboratories, Inc. Burlingame, CA, USA) containing 2.5 μg/mL DAPI (Serva, Heidelberg, Germany).

### 4.6. Image Acquisition and Analysis

The images were acquired using an Olympus FV1000 confocal system equipped with a 60×/1.35 PlanApo objective (Olympus, Tokyo, Japan) or a Zeiss Axio Imager.Z.2 wide-field epifluorescence microscope equipped with a high-sensitivity AxioCam Mrm monochromatic camera (ZEISS, Oberkochen, Germany). Image stacks were acquired by traversing from the top to the bottom of a nucleus in 200–250 nm steps. 

The photomicrographs of chromosomes in the squashed preparations were digitally colored and uniformly processed to improve contrast and brightness using ZEN 2.3 Pro (ZEISS, Oberkochen, Germany) and Photoshop CS3 (Adobe, San Jose, CA, USA). The processing of the 3-D images, including the rendering of the *Z*-stacks from a series of optical sections of the nuclei, was performed with Imaris 9.5 software (Bitplane, Zürich, Switzerland). The monochromatic images were colored using the ‘Display Adjustment’ function and merged. The same function was used to uniformly adjust the contrast and brightness of the image. Then, the ‘Contour surface’ wizard was used to construct the 3-D models of the analyzed nuclei and the territories of the added maize chromosomes inside the nuclei. These models were utilized to determine the volume of the nuclei and the maize CTs as well as to define the coordinates of the signal intensity weighted centroids as the centers of the individual or associated maize CTs, for which the surfaces were created. To determine the positioning of maize CTs in oat nuclei, the ‘Measurement Points’ function was used to measure the distance between the center of a region formed in the nucleus by a single maize CT or by associated maize CTs and the nearest edge of the nucleus (NNE-Zm). 

### 4.7. Statistical Data Analysis

Statistical analyses were performed on the acquired data in order to assess the relationship between the measured and calculated parameters, such as nuclear volume (V_nuc_), volumes of the nuclear regions occupied by maize chromatin (V_Zm_), and their sum (V_tot_), and the number of areas formed by the maize CTs inside the nucleus (N_Zm_). The V_Zm_ values inferred from the 3-D models of the nuclei were calculated as a percentage of the nuclear volume in order to allow for comparisons between nuclei of differing sizes. For V_nuc_, V_Zm_, and V_tot_, the average, median, and standard deviation values were calculated. Since the comparison between the average and median indicated positive skewness of the distribution of V_tot_, Pearson’s χ^2^ test was chosen over the Pearson linear correlation to validate the hypothesis of the independence of V_tot_ and V_nuc_. Spearman’s rank correlation coefficient was used to assess the relationship between the discrete variable N_Zm_ and continuous variables V_nuc_ and V_tot_.

The distance between the centers of maize chromatin-occupied regions and the nearest edge of the nucleus (NNE-Zm) was used to assess the positioning of maize CTs in the hybrid nuclei. All distances were normalized by dividing by the cube root of the nuclear volume, which is proportional to the radius *r* of a nucleus, assuming that the nucleus is almost spherical. The normalized distance values were grouped into 20 intervals with an interval width of 0.05 *r*. Then, the frequency distribution and probability distribution were estimated for each OMA line in both types of tissue. Pearson’s χ^2^ test was used to evaluate the relationship between the NNE-Zm distances and the size of the maize chromatin-occupied regions.

## Figures and Tables

**Table 1:** The number and ID of added maize chromosomes in the studied oat × maize addition (OMA) lines.

Genotype of the Addition Line	Symbol of the Addition Line	No. of Maize Chromosomes Added to the Oat Genome	Chromosome ID
STH 6.8636	I	1	5
STH 5.8436 b	II	2	3, 8
STH 6.8661	IVa	4	3, 9
STH 4.4690 p	IVb	4	1, 2

**Table 2:** The list of simple sequence repeat (SSR) markers used for the identification of maize chromosomes.

Maize Chromosome	SSR Marker	Maize Chromosome	SSR Marker
1	*p*-bnlg421	6	phi070
2	*p*-bnlg125	7	phi112
3	*p*-phi047	8	phi080
4	*p*-phi093, *p*-blng490	9	phi032
5	*p*-nc130	10	phi059

**Table 3:** The volumes of nuclei (V_nuc_), individual maize CTs or regions occupied by associated maize CTs (V_Zm_), and sum of the volumes of all maize CTs in a nucleus (V_tot_). The maximum (max), minimum (min), average (av), median (med), and standard deviation values (SD) were calculated for the leaf and root nuclei in each OMA line. The values of V_Zm_ and V_tot_ are shown also as a percentage of the nuclear volume.

Parameter	I Roots	I Leaves	II Roots	II Leaves	IVa Roots	IVa Leaves	IVb Roots	IVb Leaves
No. of nuclei	30	30	20	23	25	18	25	28
V_nuc _max_ [µm^3^]	1401	1849	1693	1176	1311	732	1375	857
V_nuc_min_ [µm^3^]	533	611	314	527	268	238	576	372
V_nuc_av_ [µm^3^]	1023	966	1031	795	570	463	939	544
V_nuc_med_ [µm^3^]	1050	873	1032	753	412	451	875	492
V_nuc_SD_ [µm^3^]	193	344	413	152	285	105	254	116
V_Zm_max_ [µm^3^]	36.50	33.60	32.70	37.0	18.10	42.90	67.20	47.70
V_Zm_min_ [µm^3^]	6.49	6.57	3.09	6.12	0.97	1.16	4.14	2.45
V_Zm_av_ [µm^3^]	19.31	14.55	12.99	13.67	7.20	7.65	23.96	12.16
V_Zm med_ [µm^3^]	18.35	13.45	12.00	13.00	6.46	5.43	19.30	8.57
V_Zm_SD_ [µm^3^]	7.66	6.47	7.55	5.95	3.82	6.66	15.62	9.12
V_Zm_max_ [%]	3.53%	3.33%	3.27%	5.11%	3.45%	5.86%	9.44%	8.16%
V_Zm_min_ [%]	0.61%	0.73%	0.27%	0.82%	0.14%	0.33%	0.50%	0.55%
V_Zm_av_ [%]	1.94%	1.55%	1.33%	1.73%	1.35%	1.64%	2.59%	2.24%
V_Zm_med_ [%]	1.86%	1.33%	1.15%	1.54%	1.28%	1.26%	1.94%	1.76%
V_Zm_SD_ [%]	0.80%	0.61%	0.70%	0.79%	0.66%	1.11%	1.78%	1.52%
V_tot_max_ [µm^3^]	36.50	33.60	43.60	46.10	40.85	42.90	113.40	71.50
V_tot_min_ [µm^3^]	6.49	6.57	7.57	13.60	6.67	8.01	27.81	15.70
V_tot_av_ [µm^3^]	19.75	14.55	22.08	24.97	18.44	21.26	60.38	33.45
V_tot_med_ [µm^3^]	18.35	13.45	19.42	24.50	17.94	18.59	55.00	31.83
V_tot_SD_ [µm^3^]	7.66	6.47	9.30	8.27	7.41	9.39	22.56	13.63
V_tot_max_ [%]	3.53%	3.33%	3.55%	5.39%	5.86%	7.94%	9.44%	10.56%
V_tot_min_ [%]	0.61%	0.73%	1.19%	1.89%	1.68%	2.42%	2.85%	3.18%
V_tot_av_ [%]	1.93%	1.55%	2.26%	3.17%	3.47%	4.56%	6.53%	6.15%
V_tot_med_ [%]	1.86%	1.33%	2.21%	2.90%	3.45%	4.16%	6.18%	5.62%
V_tot_SD_ [%]	0.80%	0.61%	0.68%	0.98%	1.05%	1.64%	1.83%	1.92%

## Supplementary Materials

Supplementary materials can be found at https://www.mdpi.com/1422-0067/21/12/4280/s1.

## Abbreviations

BAC	Bacterial Artificial Chromosome
CT	Chromosome Territory
FISH	Fluorescence In Situ Hybridization
GISH	Genomic In Situ Hybridization
NNE	Nearest Nuclear Edge
NOR	Nucleolar Organizing Region
OMA	Oat × Maize Addition line
SSR	Simple Sequence Repeat
